# Primary Ciliary Dyskinesia from Embryogenesis to Adulthood: Micro-CT Analysis of Stage-Dependent Upper Airway Abnormalities in *Odad3* Loss-of-Function Mouse Models

**DOI:** 10.3390/genes17091012

**Published:** 2026-08-27

**Authors:** Tiziana Orsini, Sabrina Putti, Francesco Chiani, Alessia Gambadoro, Miriam Pasquini, Olga Ermakova

**Affiliations:** 1 Institute of Biochemistry and Cell Biology (IBBC), National Research Council of Italy (CNR), Adriano Buzzati-Traverso Campus, Via Ramarini, 32, 00015 Monterotondo, Italy; sabrina.putti@cnr.it (S.P.); francesco.chiani@cnr.it (F.C.); alessia.gambadoro@cnr.it (A.G.); miriam.pasquini@cnr.it (M.P.); olga.ermakova@cnr.it (O.E.); 2 European Mouse Mutant Archive (EMMA), INFRAFRONTIER, Monterotondo Mouse Clinic, National Research Council of Italy (CNR), Adriano Buzzati-Traverso Campus, Via Ramarini, 32, 00015 Monterotondo, Italy

**Keywords:** primary ciliary dyskinesia, ciliopathy, mucociliary clearance, *Odad3*, *Ccdc151*, mouse models, conditional knockout mouse, upper airway development, micro-computed tomography, virtual histology

## Abstract

Background/Objectives: Primary ciliary dyskinesia (PCD) is a genetically heterogeneous disorder characterized by impaired ciliary function, leading to chronic airway disease. Loss-of-function mutations in *ODAD3* (*CCDC151*) represent an established cause in patients, and *Odad3*-deficient mice recapitulate key disease traits. However, the impact of *Odad3* ablation on upper airway development and function remains unexplored. This study investigated the consequences of *Odad3* disruption on upper airway structures during development and in adult animals. Methods: Constitutive (*Odad3^−^^/−^*) and inducible conditional (*Odad3^icKO^*) mouse models were analyzed alongside heterozygous and wild-type littermates during embryonic, postnatal, and adult stages. Optimized high-resolution 3D micro-computed tomography (micro-CT or µCT) combined with histology was utilized to conduct systematic genotype-phenotype evaluations, map upper airway anatomical architecture, and assess PCD disease onset. Results: Genetic dissection revealed a marked dependence of the phenotype on whether *Odad3* loss occurred during development or in adulthood. Constitutive *Odad3* deletion resulted in pervasive craniofacial remodeling and turbinate hypoplasia during embryonic stages and early postnatal development. Conversely, adult-induced conditional ablation produced localized caudal atrophy of the nasal turbinates accompanied by massive mucus accumulation, consistent with impaired mucociliary clearance and providing a 3D structural and morphological characterization of chronic rhinosinusitis-like pathology in PCD mouse models. Heterozygous *Odad3^icKO/+^* and *Odad3^+/−^* mice were phenotypically indistinguishable from wild-type controls, indicating that single-allele loss does not disrupt upper airway morphology. Conclusions: This study characterizes the structural timeline of upper airway pathology in PCD and validates the *Odad3^icKO^* model as a robust 3D structural phenotyping platform for investigating airway disease in ciliopathies. Combining targeted genetic disruption with 3D µCT virtual histology offers a powerful framework for comprehensive studies of human genetic variants and gene knockouts in mouse models of PCD.

## 1. Introduction

Primary ciliary dyskinesia (PCD; OMIM Phenotypic Series: PS244400) is a genetically heterogeneous disorder characterized by defects in the assembly, structure, or coordinated beating of motile cilia. These microscopic, microtubule-based organelles are essential for fluid homeostasis and are found on the apical surface of specialized multiciliated epithelial cells lining the respiratory tract, brain ventricles, and reproductive ducts. Additionally, motile cilia are present as solitary structures at the embryonic node, where they generate leftward fluid flow essential for establishing left-right body asymmetry and form the axonemal core of the sperm flagellum [1,2]. In the airways, ciliary activity is a central component of the mucosal defense system, as it drives mucociliary clearance (MCC). In healthy individuals, cilia propel mucus and trapped pathogens toward the pharynx through rhythmic, wave-like beats [3,4,5]. Consequently, patients with PCD exhibit severe impairment of MCC, leading to chronic sinopulmonary infections, progressive bronchiectasis, and significant upper airway disease beginning in early childhood [6,7].

At the molecular level, the rhythmic beating of the ciliary axoneme, typically organized in a (9 × 2 + 2) microtubule arrangement, is powered by specialized motor complexes known as inner and outer dynein arms (IDAs and ODAs) [8,9]. The precise assembly and attachment of these motors are regulated by the outer dynein arm docking complex (ODA-DC), where mutations in subunit-encoding genes frequently cause PCD, with *ODAD3* (formerly *CCDC151*) representing a component. In humans, nonsense and loss-of-function mutations in *ODAD3* result in the absence of ODAs and subsequent profound impairment in ciliary motility, which clinically translates into severe respiratory distress, chronic sinusitis, and laterality defects such as situs inversus totalis [3,8,9].

We previously characterized a constitutive *Odad3* knockout mouse model [10]. Phenotypic analysis confirmed that this model faithfully recapitulates the cardinal features of human primary ciliary dyskinesia (PCD) because the loss of *Odad3* function results in situs abnormalities and hydrocephalus. However, *Odad3^−/−^* animals succumb early in postnatal life, most likely due to the severity of the hydrocephalus. To circumvent this early lethality and bypass the early embryonic window of left-right axis determination, we generated a mouse model harboring a conditional *Odad3* allele that enables *Cre*-mediated deletion upon tamoxifen administration using the *Rosa26^ERT2-Cre^* driver. This strategy effectively bypasses the lethal hydrocephalic phenotype and preserves normal organ laterality, enabling late postnatal phenotypic analysis [10,11]. Although the conditional deletion of *Odad3* in adult mice has been shown to impair male fertility, recapitulating another phenotype commonly observed in patients with PCD, upper airway abnormalities have not yet been investigated in this model. While the molecular basis of *Odad3*-related PCD is well documented, the impact of ciliary dysfunction on long-term structural development and remodeling of the upper respiratory tract remains poorly understood. Building upon the previously established systemic *Cre*-mediated recombination and target deletion of the inducible *Odad3^icKO^* mouse model [10,11], the present study extends this characterization by providing a non-destructive, three-dimensional (3D) tissue- and organ-level morphometric assessment of upper respiratory tract development and remodeling across developmental stages.

Defective mucociliary clearance is a central feature of PCD and is associated with high rates of chronic sinonasal and middle ear symptoms. Interestingly, in cystic fibrosis (CF), despite a distinct genetic etiology driven by ion transport defects rather than by impaired primary ciliary motility, patients also develop chronic rhinosinusitis, with computed tomography (CT) scans often revealing agenesis of the paranasal sinuses. While this sinus hypoplasia was initially attributed to chronic inflammation, recent studies in CF animal models have demonstrated that structural sinus defects precede inflammation and may already be present at birth [12,13,14]. Similarly, several cohort studies of patients with PCD have reported a high prevalence of hypoplastic or aplastic paranasal sinuses [14,15]. Therefore, it remains unclear whether sinus abnormalities in PCD have a primary developmental origin or occur as a secondary consequence of chronic, recurrent infections.

The nasal cavity has a highly intricate skeletal organization. It is divided by the nasal septum and internally structured by scroll-like bony formations known as nasal turbinates [16]. These structures, lined by specialized respiratory and olfactory epithelia, perform several essential physiological functions. By markedly increasing the internal surface area of the nasal cavity, they contribute to warming, humidifying, and filtering inspired air while also providing a structural scaffold for the olfactory neuroepithelium [17,18]. Anatomical complexity varies significantly across species. Whereas humans possess simplified turbinates, macrosmatic rodents have a highly elaborate nasal labyrinth optimized to maximize olfactory and respiratory efficiency [19,20]. Despite their physiological importance, the dense and fragile nature of the nasal skeleton poses a significant challenge for traditional diagnostic and research methods. This technical difficulty may partly explain why upper airway defects in animal models of PCD have historically been understudied. Standard two-dimensional (2D) histological sectioning may fail to preserve or reconstruct the spatial continuity of the turbinate system, leading to fragmented interpretations of pathological remodeling [21,22].

Micro-computed tomography (µCT) has emerged as a powerful tool in developmental biology. By providing high-resolution, non-destructive three-dimensional imaging, µCT allows for the precise phenotyping of skeletal and soft tissue defects across multiple developmental stages and disease conditions [23,24]. In this study, we applied an optimized µCT-mediated virtual histology protocol to enhance X-ray contrast in soft tissues while preserving the integrity of the delicate nasal architecture. To determine whether PCD-related upper airway pathology has a primary developmental origin or arises postnatally, we systematically analyzed an allelic series of constitutive (*Odad3^−/−^*) and inducible conditional (*Odad3^icKO^*) mouse models. Specifically, we tracked the structural and morphological progression of the nasal cavity across three major temporal windows: (1) embryonic development; (2) early postnatal life; and (3) full adulthood. Embryonic analysis revealed pervasive craniofacial remodeling and turbinate hypoplasia. Analysis of the animals postnatally demonstrated structural defects characterized by ethmoturbinate mucosal degeneration and nasal passage obstruction by mucus. Adult *Odad3* ablation yielded a phenotype comparable to the postnatal phenotype, characterized by ethmoturbinate mucosal degeneration and caudal mucus accumulation. Overall, our findings indicate that impaired mucociliary clearance is a primary driver of PCD-like chronic rhinosinusitis. Ultimately, this work establishes *Odad3* loss-of-function mice as valuable experimental models and provides a powerful platform for interpreting the clinical spectrum of human genetic variants and evaluating the efficacy of emerging targeted therapies.

## 2. Materials and Methods

### 2.1. Ethics Statement

This study adhered to all relevant ethical and regulatory frameworks for animal research. All animal procedures were conducted in accordance with protocols approved by the Ethical and Scientific Commission of the Veterinary Health and Welfare Department of the Italian Ministry of Health (protocol approval reference: N:0000688 21 March 2008). The study also complied with established guidelines for animal welfare set forth by Italian laws and regulations, which are aligned with the relevant European Union directives (86/609/EEC and 2010/63/EU).

### 2.2. Animal Model

All mice utilized in this study were of the C57BL/6N background and were bred and maintained at the European Mouse Mutant Archive (EMMA) facility in Monterotondo, Italy, under specific pathogen-free (SPF) conditions. Animals were housed under strictly controlled environmental conditions, including a temperature of 21 °C, a 12 h light/dark cycle (lights on 7 a.m. to 7 p.m.), and ad libitum access to food and water, with mice grouped 3–5 per cage by litter and sex. The *Odad3^tm1b/+^* line (previously *Ccdc151^tm1b/+^*) was originally generated by the IKMC consortium at Monterotondo (details in [10,11]). Genotyping of the *Odad3^tm1b^* allele was performed using specific primers (*Odad3*-F: AGAGCCCTGGATCTTAACTGCTGA; *Odad3*-R: TCCAAGTCATGCAGAGCTGGGATT; Frt-Rev: CCTTCCTCCTACATAGTTGGCAGT), yielding a PCR product of 307 bp for the wild-type (WT) allele and 270 bp for the *Odad3^tm1b^* allele. Furthermore, the generation of mice harboring the conditional knockout allele (*Odad3^icKO^*) is detailed in the aforementioned publications, and this allele was genotyped using the same set of primers, resulting in WT and conditional knockout bands of 305 bp and 481 bp, respectively. The cohorts used for Cre-mediated induction were generated such that the experimental group carried homozygous *Odad3* conditional alleles, while the control group carried heterozygous *Odad3* conditional alleles. Both groups were heterozygous for the *ROSA26^ERT2-Cre^* allele (MGI:3764519). *Cre*-recombinase expression was induced in 4-month-old mice via daily intraperitoneal (i.p.) injections of tamoxifen (1 mg/day; Sigma-Aldrich, St. Louis, MO, USA, T5648), dissolved in corn oil (Sigma-Aldrich, St. Louis, MO, USA, C8267) at a concentration of 10 mg/mL for five consecutive days. Nasal structure analysis was performed 4 months after tamoxifen injections.

### 2.3. Optimized Tissue Processing Protocol: Enhancing Soft Tissue Contrast for Micro-CT and Histology

To enable complementary high-resolution µCT analysis and subsequent histological evaluation, we optimized the tissue processing protocol. This method was specifically designed to enhance the inherent X-ray contrast of soft, connective, and epithelial tissues while eliminating the high X-ray attenuation of calcified bone (via decalcification), ensuring the tissue is suitable for both imaging and sectioning.

### 2.4. Processing of Calcified Skulls (Adult/Postnatal Samples)

All skulls were initially fixed in 4% paraformaldehyde (PFA) overnight (O.N.) at 4 °C to preserve tissue architecture prior to chemical decalcification. Decalcification was performed by immersion in an 8% EDTA/50 mM Tris solution (pH 8.5) at room temperature with agitation for approximately two weeks. This extensive aldehyde fixation and prolonged chelation treatment were essential to remove the highly attenuating bone matrix for 3D structural µCT and histological sectioning, although such processing precludes quantitative local tissue protein/RNA extractions (e.g., Western blot, qPCR) or epitope-sensitive immunohistochemistry on these specific imaging cohorts. Following decalcification, the samples were extensively washed with phosphate-buffered saline (1× PBS) to remove debris and post-fixed in 4% PFA O.N. to ensure optimal tissue preservation. The subsequent steps of dehydration, clearing, and paraffin infiltration followed the established protocol described by Ermakova et al. (2018) [25]: samples were dehydrated through a graded ethanol series (50% and 70% overnight, 95% for 30 min, and 100% for 1 h), cleared in xylene (45 min), and sequentially infiltrated with a 1:1 xylene/paraffin solution (45 min at 56 °C) and pure paraffin wax (overnight at 56 °C). Final embedding was performed in molds for solidification (1 h). Crucially, µCT imaging was performed on the resulting paraffin blocks prior to sectioning, ensuring the preservation of the native 3D spatial orientation.

### 2.5. Processing of Embryos (E12.5 Samples)

E12.5 embryos were carefully dissected and staged according to standard morphological criteria prior to processing [24,25]. These samples, which exhibit minimal bone mineralization, were fixed in 4% PFA overnight at 4 °C immediately following washing in 1× PBS. As significant calcified structures were absent at this developmental stage, the decalcification step was logically omitted to preserve soft tissue integrity. The subsequent dehydration, clearing, and paraffin infiltration procedures were performed identically to the skull processing protocol.

### 2.6. High-Resolution µ-CT Analysis

Three-dimensional micro-computed tomography (micro-CT or µCT) images were acquired using a high-resolution imaging system (SkyScan 1172G, Bruker, Kontich, Belgium) coupled with an L7901-20 Microfocus X-ray Source (Hamamatsu Photonics, Hamamatsu, Japan). Volume acquisition for the skulls was performed using a camera pixel size of 9 µm, a camera binning of 2 × 2, a tube voltage of 39 kV, a tube current of 240 µA, and an exposure time of 650 ms, resulting in a final isotropic voxel size of 13.56 µm. Conversely, for embryos, volume acquisition utilized a camera pixel size of 9 µm, a camera binning of 2 × 2, a tube voltage of 39 kV, a tube current of 240 µA, and an exposure time of 400 ms, resulting in a 4.88 µm isotropic voxel size. Subsequently, tomographic datasets were reconstructed using the built-in NRecon SkyScan Software (version: 1.6.6.0; Bruker). For visualization and qualitative assessment, the 3D volumes were analyzed using CTvox v. 2.5 (Bruker), while morphometric quantification of ethmoturbinate thickness was performed using Bruker micro-CT Analyser Version 1.13 software after datasets were accurately reoriented to identify the optimal cutting plane for ethmoturbinate visualization. Finally, for statistical analysis, differences between the experimental groups were analyzed using unpaired Student’s *t*-tests (with data expressed as mean ± SEM), and all data analysis was conducted using GraphPad Prism 6 software, with statistical significance set at *p* < 0.05. Morphometric measurements were focused on adult mice, as they provided a stabilized pathological framework for quantification of turbinate atrophy and mucosal thickness. Embryonic (E12.5) and postnatal (P11) stages were primarily utilized for a 3D qualitative assessment to identify the ontogenetic onset of the phenotype.

### 2.7. Histological Validation

Following µCT acquisition, the same paraffin blocks were sectioned serially at 8 µm thickness using a microtome. The resulting cross-sections were prepared for standard Hematoxylin and Eosin (H&E) staining [26] by overnight heating at 45 °C, followed by dewaxing with xylene and rehydration through a graded ethanol series. The staining procedure involved two minutes in hematoxylin, differentiation with 1% hydrochloric ethanol (30 s), bluing with 1% ammonia (30 s), and two minutes of eosin counterstaining. Sections were then dehydrated, cleared twice with xylene (5 min each), and mounted using neutral mounting medium. Bright-field images of the complete cross-sections were acquired using a motorized scope (LMD 7000, Leica Microsystems) equipped with 20× and 40× dry objectives (HC PL FLUOTAR 20× NA: 0.4, Leica Microsystems, Wetzlar, Germany). Images were subsequently imported into ImageJ version 1.54g (National Institutes of Health, Bethesda, MA, USA) for high-resolution correlation with the corresponding µCT virtual sections.

## 3. Results

### 3.1. A µCT-Based Virtual Histology Workflow Enables Standardized Mapping of the Adult Mouse Nasal Cavity

**Figure 1:**
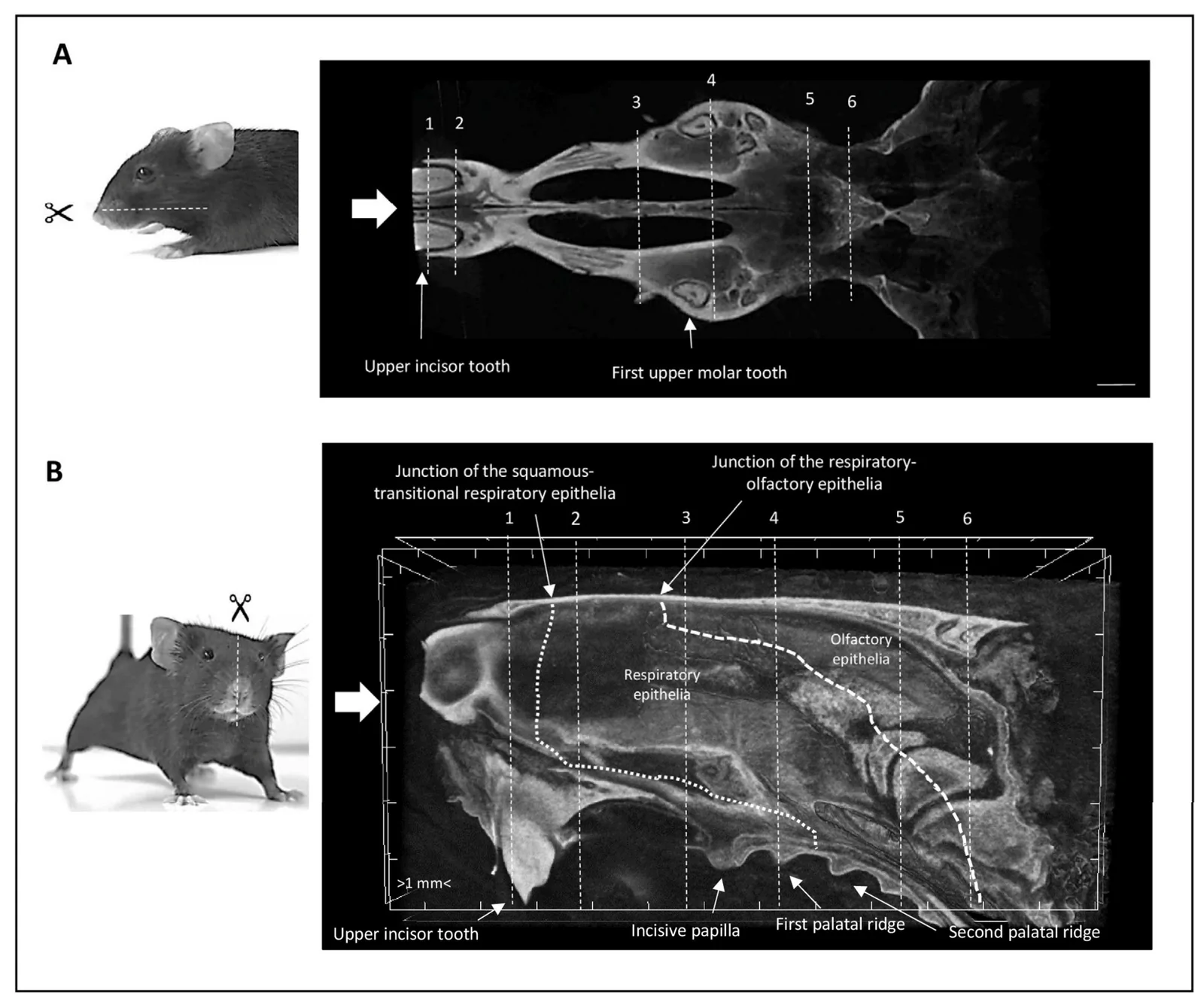
Transverse and sagittal views of the dorsal buccal cavity and nasal passages in a non-treated WT mouse. (**A**) Tomographic transverse section of the dorsal buccal cavity in WT mice, with numbering of the six main cutting planes for the identification of the corresponding principal nasal passages, as shown in Figure 2 (coronal view). (**B**) Tomographic sagittal section of the dorsal buccal cavity and nasal passages in WT mice, which shows the corresponding numbering of the cutting planes indicated in (**A**) and represented in Figure 2; scale bar 1 mm. Scissors and arrow indicate the sectioning plane.

**Figure 2:**
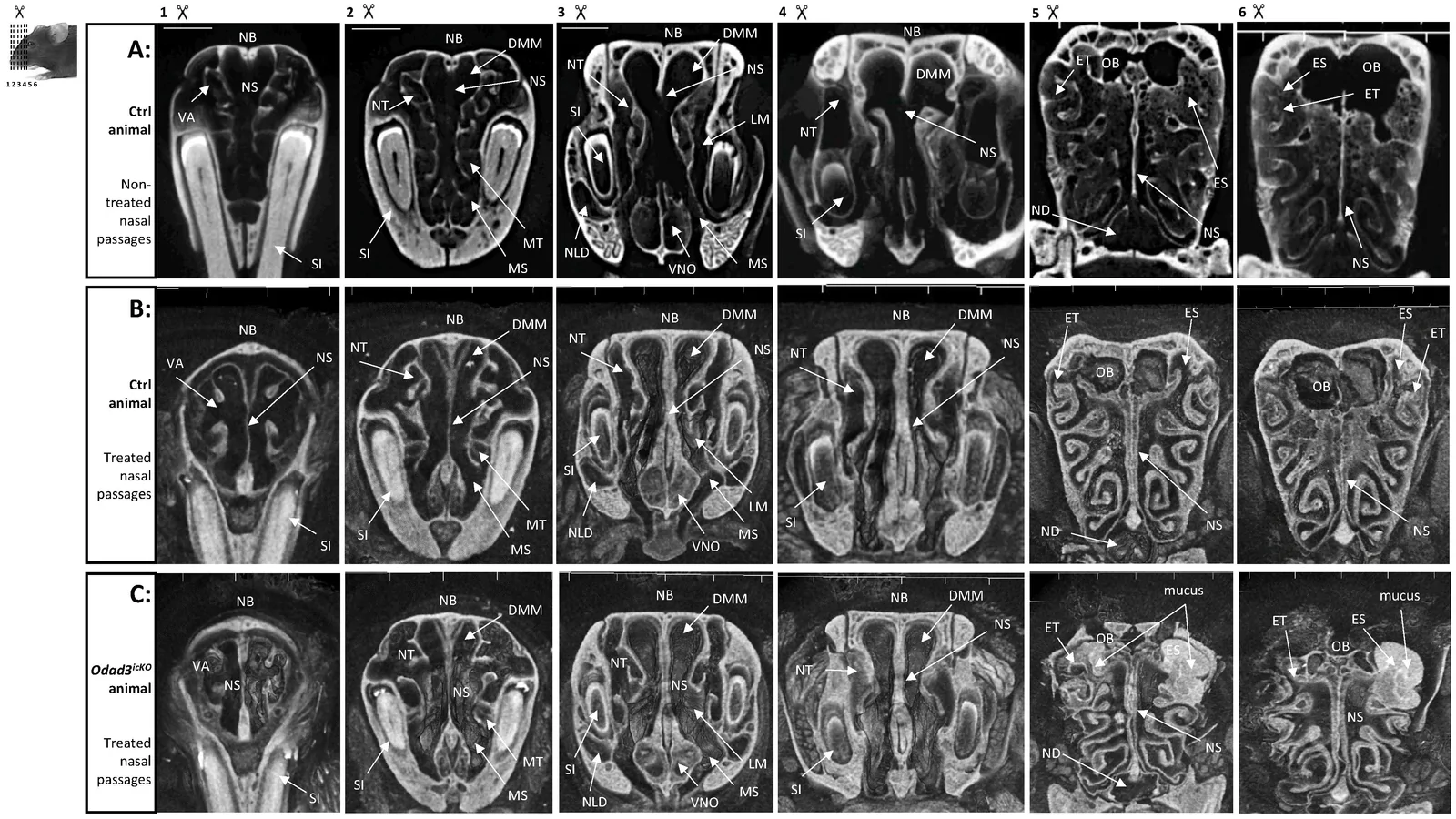
Six principal coronal section levels of the adult mouse nasal cavity. (**A**) Tomographic coronal sections of a non-decalcified nasal cavity from a control mouse (tamoxifen-induced heterozygous *Odad3^icKO/+^* littermate, indistinguishable from WT); scale bar 1 mm. (**B**) Tomographic coronal sections of the same nasal cavity following tissue processing (decalcification and paraffin embedding); scale bar 1 mm. (**C**) Tomographic coronal sections of a processed (decalcified and paraffin-embedded) nasal cavity from a homozygous mutant *Odad3^icKO^* mouse; scale bar 1 mm (*N* = 4 per genotype). Nasal bone (NB); olfactory bulb (OB); ethmoturbinate (ET); ethmoid sinus (ES); nasal septum (NS); maxilloturbinate (MT); maxillary sinus (MS); dorsal medial meatus (DMM); nasoturbinate (NT); atrioturbinate (VA); lateral meatus (LM); nasolacrimal duct (NLD); nasopharyngeal duct (ND); vomeronasal organ (VNO); superior incisor (SI). Scissors icons, dashed lines, and numbers indicate the location, orientation, and sequence of the sectioning planes.

To investigate the structural effects of *Odad3* gene ablation on the upper respiratory tract, we applied a tailored three-dimensional (3D) micro-computed tomography (µCT) imaging protocol. This approach enables the simultaneous visualization of both bony structures and soft tissues by exploiting differences in X-ray attenuation following specialized sample processing. Specifically, the methodology combines bone decalcification, paraffin embedding, and high-resolution volumetric µCT imaging to enhance contrast among the soft, connective, and epithelial tissues associated with the nasal cavity. We first established this virtual histology approach by constructing a standardized 3D virtual atlas of the murine upper airways and defining six principal coronal planes suitable for the analysis of PCD mouse models (Figure 1 and Figure 2).

This mapping used established anatomical landmarks within the palatal cavity as reference points, following the standard methodology described by Mery et al. (1994) [27]. Anatomical landmarks within the wild-type (WT) adult palatal cavity were used to anchor the six reference coronal planes (Figure 1). Specifically, the transverse tomographic section (Figure 1A) shows the positions of the incisors and anterior molars, which define the locations of planes 1, 2, and 4; planes 1 and 2 are situated anterior and posterior to the incisors, respectively, whereas plane 4 lies immediately caudal to the anterior molars. The central sagittal section (Figure 1B) illustrates how the incisive papilla and palatal ridges were used to define the remaining planes (3, 5, and 6). All corresponding coronal views are sequentially displayed in Figure 2.

These six standard coronal planes provide an anatomical reference for the principal nasal passages in adult murine models, detailing their underlying bony and cartilaginous frameworks. To optimize soft-tissue contrast and enable subsequent histological correlation on the same specimens, we implemented a tissue processing protocol based on EDTA decalcification followed by paraffin embedding prior to µCT scanning. To assess the effect of this sample preparation procedure, we compared µCT images obtained from standard, non-decalcified control skulls (Figure 2A) with those obtained after decalcification and paraffin embedding (Figure 2B). Imaging of non-decalcified samples clearly delineated highly attenuating structures, such as the nasal bone (NB), nasal septum (NS), and the bony cores of the turbinates (ethmoturbinate—ET, maxilloturbinate—MT, nasoturbinate—NT, atrioturbinate—VA) but provided limited contrast of the overlying soft tissues. In contrast, tomographic volumes obtained after decalcification and paraffin embedding permitted the simultaneous visualization of the skeletal framework and the spatial organization of the respiratory and olfactory epithelia across all six coronal planes (Figure 2B). The improved soft-tissue contrast was particularly evident in the complex ethmoturbinate region represented by planes 5 and 6. It also enabled the identification of adjacent anatomical structures, including the maxillary sinus (MS), ethmoid sinus (ES), nasolacrimal duct (NLD), nasopharyngeal duct (ND), and vomeronasal organ (VNO).

### 3.2. Adult-Induced Odad3 Deletion Causes Caudal Upper-Airway Remodeling and Luminal Obstruction

To ensure appropriate controls in the inducible models, tamoxifen-induced heterozygous mice (*Odad3^icKO/+^*) were utilized as the primary control group (Figure 2A,B, Supplementary Video S4). Preliminary morphometric assessment indicated that these induced heterozygous controls were phenotypically and structurally indistinguishable from untreated WT littermates and constitutive heterozygous (*Odad3^+/−^*) mice (Supplementary Videos S1 and S3). We next used high-resolution µCT to characterize the structural consequences of adult *Odad3* deletion while circumventing the early postnatal lethality observed in constitutive *Odad3^−/−^* mutants. Conditional deletion of the *Odad3* gene was induced in adult mice via tamoxifen injections, which activate the expression of the *ER^T2^-Cre* recombinase placed under the control of the ubiquitous *Rosa26* promoter (*Odad3^icKO^*). Following a 4-month post-induction period, the skulls were processed using our sample preparation protocol and imaged by µCT. We observed pronounced alterations in the upper airway architecture of the *Odad3^icKO^* but not in the *Odad3^icKO/+^* (Figure 2C, Supplementary Video S2). A detailed rostral-to-caudal comparison between the control passages (Figure 2B) and their *Odad3^icKO^* counterparts (Figure 2C) under identical imaging parameters revealed a spatially distinct pattern of pathology. No overt structural abnormalities were observed in the anterior regions corresponding to planes 1–4 (Figure 2B versus Figure 2C). In contrast, severe tissue remodeling was detected in the caudal regions represented by planes 5 and 6. Conditional *Odad3* ablation was associated with pronounced thinning and atrophy of the ethmoturbinates, extensive mucosal degeneration, and narrowing of the corresponding nasal passages. Furthermore, the nasal cavities and meati contained abundant luminal material consistent with mucus accumulation, resulting in extensive obstruction of the caudal nasal passages. (Figure 2C, planes 5 and 6). Together, these morphological changes were consistent with severe chronic rhinosinusitis-like upper-airway pathology developing within four months of adult *Odad3* deletion. Consistent with successful *Cre*-mediated induction, this phenotype was observed in all examined homozygous *Odad3^icKO^* mice, whereas tamoxifen-treated heterozygous controls (*Odad3^icKO/+^)* retained completely normal upper-airway architecture.

### 3.3. Correlative Histology and µCT Morphometry Confirm Ethmoturbinate Atrophy and Mucosal Degeneration in Odad3^icKO^ Mice

**Figure 3:**
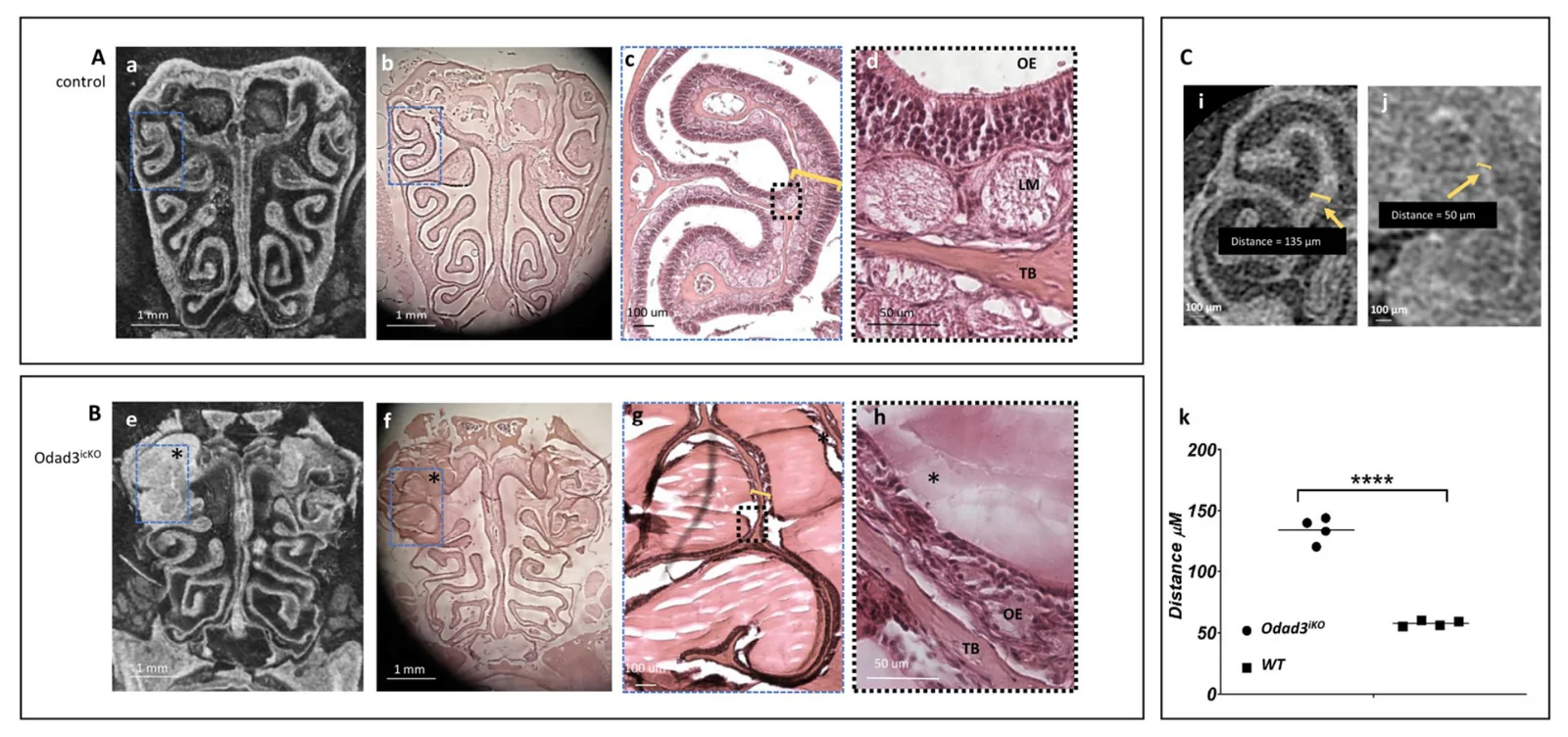
Structural analysis of ethmoturbinates in control and *Odad3^icKO^* mice. (**A**) Representative coronal µCT virtual histology (**a**) and corresponding hematoxylin and eosin (H&E) histological sections (**b**–**d**) of the olfactory turbinates in *Odad3^icKO/+^* control mice. The blue squares in (**a**,**b**,**e**,**f**) indicate the areas enlarged in (**c**,**g**). The dashed black squares in (**c**,**g**) mark the regions shown at higher magnification in (**d**,**h**). Scale bars: 1 mm (**a**,**b**); 100 µm (**c**); 50 µm (**d**). (**B**) Coronal µCT virtual histology (**e**) and H&E tracking (**f**–**h**) of degenerate olfactory turbinates in *Odad3^icKO^* mice. Scale bars: 1 mm (**e**); 100 µm (**f**,**g**); 50 µm (**h**). (**C**) Morphometric µCT evaluation of ethmoturbinate structure thickness of control (**i**) and *Odad3^icKO^* (**j**); yellow square brackets and arrows indicate the measured thickness of the ethmoturbinate framework. Scale bars: 100 µm (**i**,**j**). Asterisks (*) indicate pathological mucus accumulation. Graphical quantification (**k**) demonstrates a severe reduction in ethmoturbinate thickness in *Odad3^icKO^* animals compared to controls. (horizontal lines represent group means). Data are presented as mean ± SEM (*N* = 4 per genotype); **** *p* ≤ 0.0001 by an unpaired two-tailed Student’s *t*-test. Abbreviations: olfactory epithelium (OE); lamina propria (LM); turbinate bone (TB).

To validate the structural and obstructive alterations detected via 3D volumetric imaging, a correlative histopathological analysis was conducted at the precise anatomical levels identified via µCT. We focused our assessment on coronal plane 5, corresponding to the caudal regions shown in Figure 2B for controls and Figure 2C for *Odad3^icKO^* mice, where the three-dimensional rendering revealed the most pronounced tissue remodeling and airway occlusion (Figure 3A versus Figure 3B). Multi-planar reformatting allowed the µCT volumes to be reoriented along different spatial axes and aligned with the physical sectioning plane of the corresponding paraffin blocks. By aligning the virtual datasets with the palatal landmarks defined in Figure 1, histological sections from plane 5 in adult control nasal cavities (Figure 3b–d) showed close anatomical correspondence with the virtual µCT sections (Figure 3a), reproducing the organization of the skeletal framework, epithelial linings, and patent airspaces.

Histological sections from *Odad3^icKO^* mice corroborated the abnormalities detected by µCT, confirming severe mucosal degeneration and extensive obstruction of the respiratory passages (Figure 3f–h). At higher magnification, mutant ethmoturbinates displayed marked disruption and thinning of the olfactory epithelium (Figure 3b–d versus Figure 3f–h). The lamina propria (LM) also exhibited structural disorganization, thinning, and cellular degeneration (Figure 3g,h). Furthermore, the ethmoturbinate region was surrounded by abundant mucoid luminal material (Figure 3e,f,h), consistent with stagnant mucus obstructing the nasal meati.

To quantify the extent of ethmoturbinate remodeling, we used multi-planar reformatting to perform morphometric measurements along consistent anatomical axes. This approach enabled reproducible evaluation of the convoluted ethmoturbinate structures while reducing variability associated with differences in physical sectioning orientation.

Total ethmoturbinate thickness was defined as the combined cross-sectional width of the olfactory epithelium (OE), lamina propria (LM), and central turbinate bone (TB) core. Morphometric analysis revealed marked thinning of the ethmoturbinates in adult *Odad3^icKO^* mice compared with controls (Figure 3i,j). Quantification confirmed a highly significant reduction in total ethmoturbinate thickness (Figure 3i,j). These findings support the presence of severe ethmoturbinate atrophy following adult *Odad3* deletion (Figure 3k).

### 3.4. Constitutive Odad3 Loss Causes Widespread Upper-Airway Abnormalities at the Postnatal Stage (P11)

**Figure 4:**
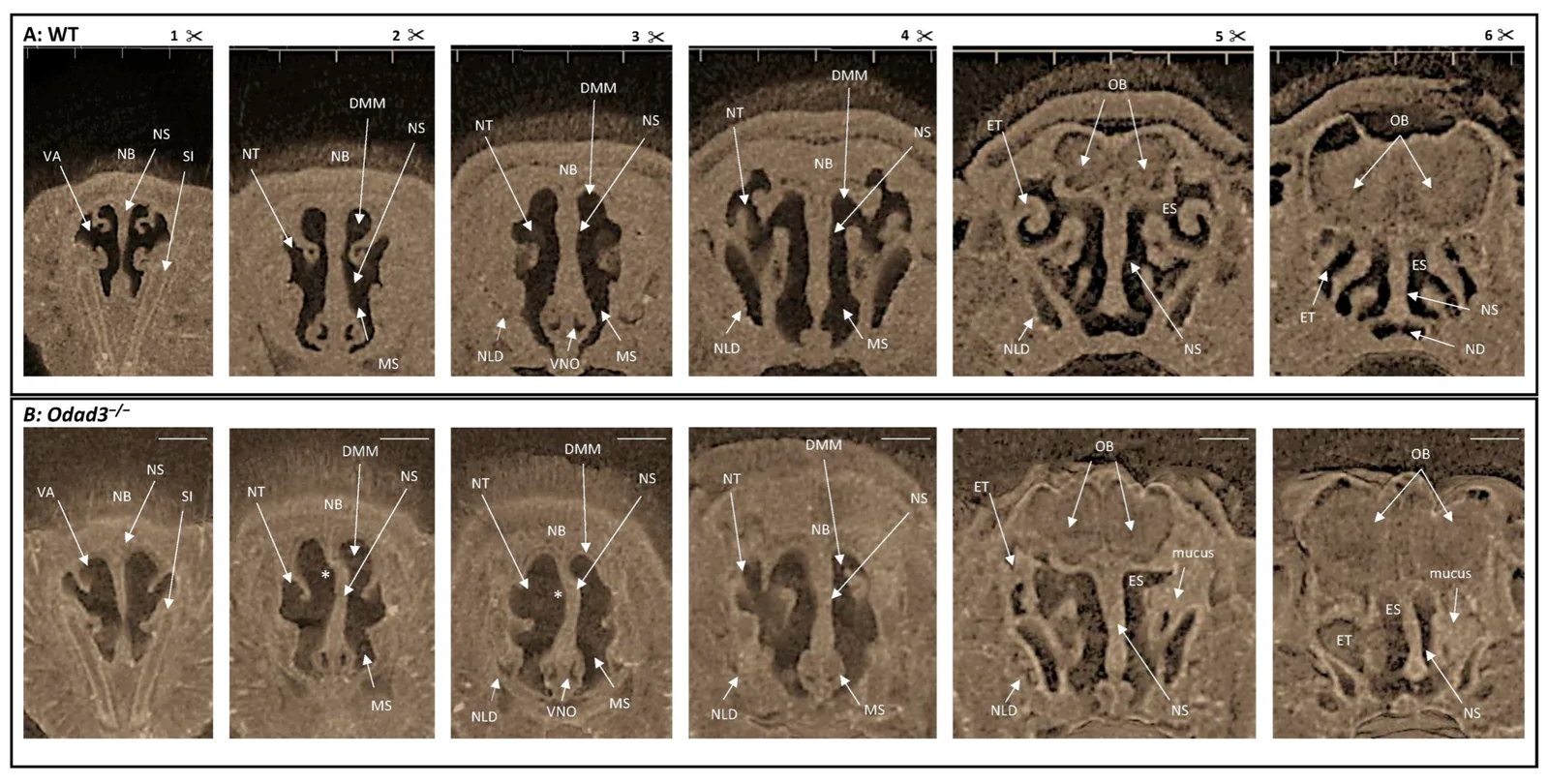
Postnatal development of nasal structures in constitutive knockout (*Odad3^−/−^*) mice at postnatal day 11 (P11). (**A**) Representative coronal µCT virtual histology sections of nasal passages across planes 1–6 in wild-type (WT) pups. Scale bar: 1 mm. (**B**) Representative coronal µCT virtual histology sections of nasal passages across planes 1–6 in *Odad3^−/−^* pups. Scale bar: 1 mm. The asterisk (*) indicates pronounced septal deviation. *N* = 4 per genotype. Abbreviations: nasal bone (NB); olfactory bulb (OB); ethmoturbinate (ET); ethmoid sinus (ES); nasal septum (NS); maxillary sinus (MS); dorsal medial meatus (DMM); nasoturbinate (NT); atrioturbinate (VA); nasolacrimal duct (NLD); nasopharyngeal duct (ND); vomeronasal organ (VNO); superior incisor (SI). Scissors icons and numbers indicate the location and sequence of the sectioning planes.

To investigate the upper-airway phenotype during early postnatal development, we performed µCT analysis on constitutive knockout (*Odad3^−/−^*) mice at postnatal day 11 (P11; Figure 4), immediately before the period of early lethality previously reported for this model [10]. P11 skulls were processed using the same contrast-enhancing protocol applied to adult samples. The reconstructed datasets were then spatially reoriented according to the anatomical alignment strategy established for adult specimens (Figure 1 and Figure 2), allowing the six reference coronal planes to be reproduced across the postnatal nasal cavities (Figure 4).

Comparison of WT (Figure 4A, Supplementary Video S5) and *Odad3^−/−^* pups (Figure 4B, Supplementary Video S6) revealed several abnormalities resembling those observed in adult conditional mutants. These included ethmoturbinate mucosal degeneration, disorganization of the olfactory epithelium, and obstruction of the nasal passages by accumulated luminal material consistent with mucus. However, the phenotype in constitutive *Odad3^−/−^* mice was more widely distributed than that observed following adult-induced deletion. Whereas adult *Odad3^icKO^* mice showed predominantly caudal abnormalities involving planes 5 and 6, P11 *Odad3^−/−^* pups displayed structural alterations across all six analyzed coronal planes. Constitutive mutants also exhibited marked deviation of the nasal septum (Figure 4B, NS*) and pronounced hypoplasia of the supporting structures of the nasal cavity, which may reflect, at least in part, mechanical distortion secondary to the severe hydrocephalus present at this developmental stage. These abnormalities were already evident in the anterior regions represented by planes 1–4. Thus, constitutive loss of *Odad3* was associated with widespread structural abnormalities throughout the nasal cavity by early postnatal life.

### 3.5. Nasal Cavity Abnormalities Are Detectable in Odad3^−/−^ Embryos at E12.5

**Figure 5:**
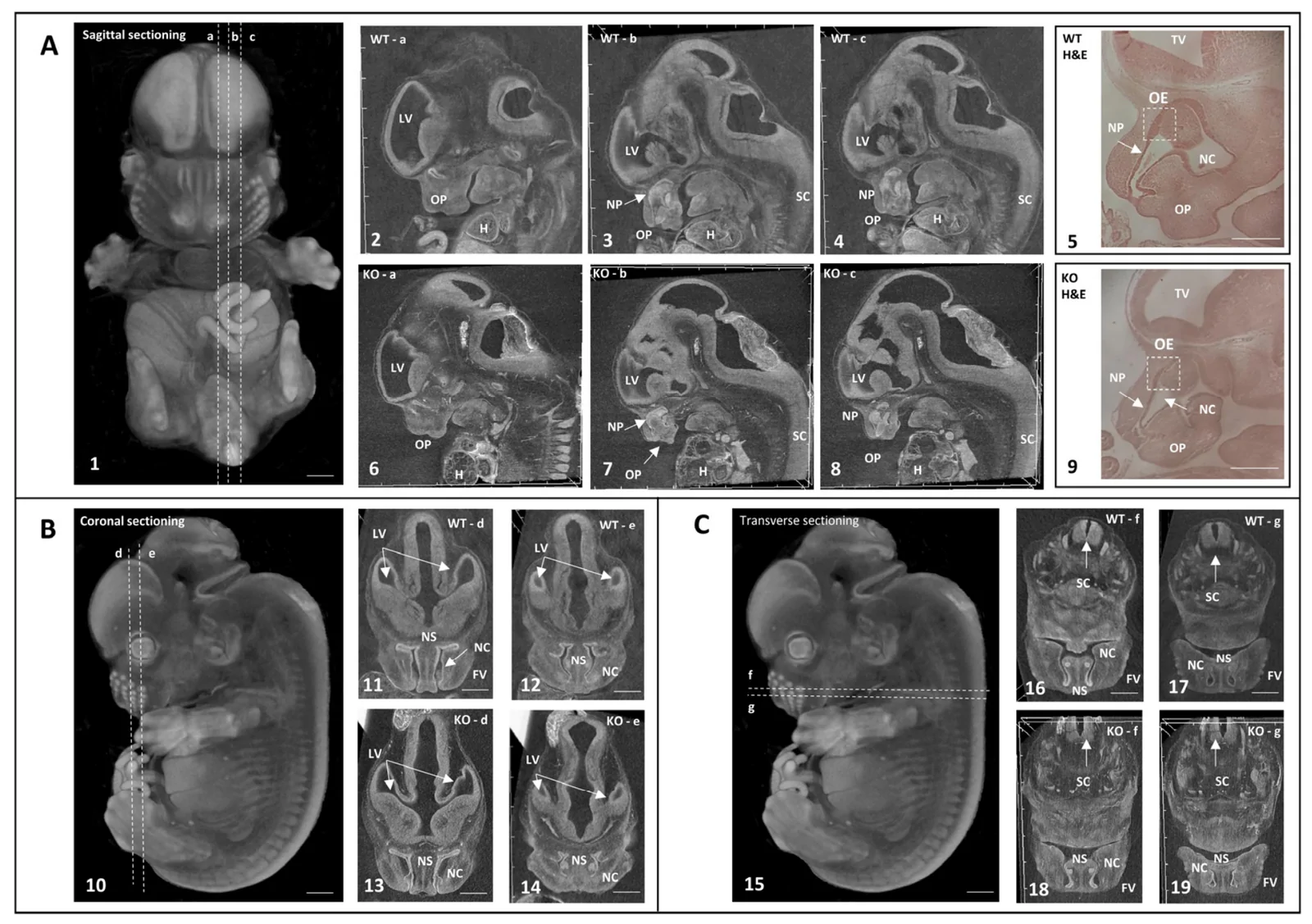
Micro-CT imaging and multi-planar virtual histology of wild-type and *Odad3*^−/−^ embryos at stage E12.5. (**A**) Sagittal tomographic workflow showing 3D volume orientation (panel 1) across three progressive depths (a–c) for wild-type (WT; panels 2–4) and *Odad3^−/−^* embryos (KO; panels 6–8), alongside corresponding H&E histological validation for WT (panel 5) and *Odad3^−/−^* (panel 9). Dashed lines labeled with letters indicate the sectioning depths across the respective anatomical planes (a–c for sagittal, d,e for coronal, and f,g for transverse). White dotted squares highlight structural details of the olfactory epithelium. Scale bar: 500 µm. (**B**) Coronal tomographic orientation (panel 10) across two reference depths (d,e) comparing WT (panels 11–12) with *Odad3^−/−^* embryos (panels 13–14). Scale bar: 500 µm. (**C**) Transverse tomographic orientation (panel 15) across two reference depths (f,g) comparing WT (panels 16–17) with *Odad3^−/−^* embryos (panels 18–19). Scale bar: 500 µm; *N* = 4 per genotype. Abbreviations: olfactory epithelium (OE); primitive nasal cavity (NC); cartilage primordium of the nasal septum (NS); lateral ventricle (LV); follicles of vibrissae (FV); nasal pit (NP); olfactory nasal placode (OP); heart (H); spinal cord (SP).

To determine whether nasal abnormalities were already present during embryonic development, we extended the analysis to E12.5 embryos (Figure 5). This stage coincides with the emergence of recognizable nasal cavity primordia, including the nasal pits, olfactory placode, turbinate primordia, and cartilaginous nasal septum [21]. The nascent epithelia are also clearly discernible, particularly the olfactory epithelium lining the posterior regions of the embryonic nasal placode. Notably, because the embryonic craniofacial skeleton at E12.5 consists entirely of unmineralized cartilage primordia rather than mature bone, our contrast-enhancing tissue processing workflow facilitated high-resolution µCT imaging directly within paraffin-embedded samples without requiring a decalcification step.

Leveraging the multi-planar versatility of 3D volumetric analysis, we performed virtual histology of the embryonic nasal cavities along the sagittal (Figure 5A, panels 1–9), coronal (Figure 5B, panels 10–14), and transverse (Figure 5C, panels 15–19) axes. To ensure thorough anatomical sampling, multiple progressive depths were systematically analyzed for each orientation, spanning three cutting levels for the sagittal axis (a–c) and two levels each for the coronal (d,e) and transverse planes (f,g). Crucially, this non-destructive imaging approach preserved sample integrity for subsequent conventional histological validation via hematoxylin and eosin (H&E) staining (Figure 5A, panels 5 vs. 9, WT vs. *Odad3^−/−^*, respectively). A systematic comparative analysis between WT (Supplementary Video S7) and *Odad3^−/−^* (Supplementary Video S8) embryos revealed structural differences during early nasal cavity development. Mutant embryos showed reduced development of the olfactory nasal placode and hypoplasia of the ethmoturbinate, maxilloturbinate, and nasoturbinate primordia. These differences were visible across sagittal, coronal, and transverse orientations (compare mutant panels 6–8, 13–14, and 18–19 with their respective WT controls, panels 2–4, 11–12, and 16–17). Histological analysis supported the µCT observations and showed a thinner olfactory epithelium lining the primitive dorsal region of the nasal cavity in *Odad3^−/−^* embryos (Figure 5A, panels 5 and 9). These observations indicate that structural abnormalities are detectable in constitutive *Odad3^−/−^* embryos by E12.5, although further quantitative analysis is required to determine whether this represents a localized nasal defect or to rule out a generalized developmental delay. Importantly, in contrast to the postnatal and adult phenotypes, no luminal material accumulation or mechanical obstruction was detected at E12.5, indicating that mucus accumulation and airway blockage emerge strictly after birth.

Taken together, these cross-sectional analyses identify distinct stage-dependent upper-airway consequences of *Odad3* loss. Constitutive disruption was associated with early nasal developmental abnormalities detectable at E12.5 and widespread structural defects at P11. By contrast, deletion induced after completion of development produced a predominantly caudal phenotype characterized by ethmoturbinate atrophy, mucosal degeneration, and luminal obstruction. These findings distinguish an early developmental component associated with constitutive *Odad3* loss from an acquired upper-airway phenotype following adult gene ablation.

## 4. Discussion

The integration of high-resolution µCT-based virtual histology with conventional histological validation provides a robust approach for the three-dimensional assessment of craniofacial and upper-airway anatomy [28]. By leveraging this non-destructive 3D imaging approach, we ensured high-fidelity anatomical registration and precise orientation across different genotypes and developmental stages, overcoming the inherent limitations of traditional two-dimensional (2D) sectioning, which often suffers from spatial data loss. We applied µCT imaging to study the normal anatomy of the nasal cavities and pathological remodeling in an allelic series of the *Odad3* loss-of-function mouse models of PCD.

µCT imaging studies have previously been used to evaluate mucociliary clearance (MCC) efficiency and mucus accumulation in the upper airways of a PCD mouse model carrying a conditional deletion of the dynein axonemal intermediate chain 1 protein (*Dnaic1*) gene. In those studies, the µCT X-ray imaging was performed on untreated animal heads [23], and mucus accumulation in the nasal cavities was inferred indirectly from the absence of airspace, as the air in affected regions, particularly the ethmoturbinate region and maxillary sinuses, was replaced by mucus. In our approach, we implemented a decalcification protocol combined with paraffin embedding prior to X-ray imaging. This procedure significantly enhances contrast in soft tissues within the nasal cavities, enabling enhanced contrast-based visualization of luminal material accumulation as well as the direct measurement of ethmoturbinate thickness. Consequently, this protocol allows for a quantitative assessment of mucosal epithelial degeneration at sufficient resolution for tissue-level morphometry and histological co-registration. Together, our results highlight the utility of this µCT-based approach for the quantitative evaluation of upper airway defects in PCD mouse models and pathology progression directly in 3D.

Here, we report that adult-induced deletion of the *Odad3* gene leads to a severe chronic rhinosinusitis (CRS)-like pathology after a 4-month post-ablation period. This is in agreement with the studies performed by Ostrowski et al. (2010) [29], who demonstrated that the conditional deletion of the *Dnaic1* gene, a crucial component of the outer dynein arm, using a similar induction protocol (*Rosa26^ERT2-Cre^* driver activated by tamoxifen), leads to a reduction in MCC at 1 month post-induction and a complete loss of MCC after 3 months. Animals lacking MCC developed full-blown CRS with mucus accumulation, a loss of normally differentiated epithelial cells at the sites of mucus stagnation, and a thinning of the lamina propria (LM), confirming these features as the most common manifestations of an MCC defect. Overall, we have demonstrated that the *Odad3^icKO^* model recapitulates similar histopathological features reported following inducible *Dnaic1* ablation, including luminal material accumulation, epithelial degeneration, and ethmoturbinate atrophy.

Clinical symptoms of PCD frequently manifest immediately after birth as respiratory distress in newborns via mechanisms that remain poorly understood. In addition, common findings in infants and children with PCD include daily nasal congestion (rhinitis) and a year-round wet cough occurring soon after birth [30]. We therefore asked whether early postnatal constitutive *Odad3* knockout animals already exhibit upper-airway abnormalities. Indeed, luminal material accumulation was widespread, coinciding with severe hypoplasia of the olfactory epithelium detected at postnatal day 11 (P11). These data are in agreement with another murine PCD model carrying a knockout of the radial spoke head 1 homolog (*Rsph1*) protein, where mucus accumulation was detected as early as postnatal day 3 [31]. Thus, *Odad3^−/−^* animals recapitulate early upper-airway manifestations associated with PCD early in life.

Regarding the widespread phenotype observed in *Odad3^−/−^* neonates, the marked septal deviation and upper-airway distortion at P11 warrant careful consideration. Given the early onset and severity of hydrocephalus in constitutive mutants, increased intracranial pressure and cranial vault remodeling may exert physical forces that mechanically deform the developing nasal septum and surrounding facial structures. Consequently, while adult-induced deletion isolates the direct effects of *Odad3* loss in the upper respiratory tract, the early postnatal phenotype likely reflects a combination of primary structural alterations and secondary mechanical strain driven by hydrocephalus.

Our findings suggest that constitutive *Odad3* loss is associated with an early developmental alteration of nasal cavity morphogenesis. Importantly, multiciliated epithelial differentiation and functional mucociliary clearance in the respiratory tract are not yet established at E12.5; therefore, the observed turbinate hypoplasia cannot be attributed to secondary mechanical or obstructive processes (such as mucus accumulation or physical blockage). This early embryonic manifestation raises an intriguing mechanistic question. Specifically, the observation of early nasal hypoplasia and stunted turbinate growth as early as E12.5 raises the possibility that early nodal ciliary dysfunction may impact craniofacial development. We hypothesize that this early developmental defect stems from the crucial role of *Odad3* in the motile monocilia of the embryonic node during gastrulation (embryonic days E7.5–E8.0). Disruption of nodal ciliary motility is known to perturb left–right patterning through altered Nodal–Lefty signaling, accounting for the situs inversus observed in constitutive mutants. In contrast, temporal conditional ablation bypasses this early gastrulation window, preserving normal organ laterality (situs solitus) while selectively enabling the isolation of post-developmental respiratory pathology. Whether early nodal ciliary function requirements and associated laterality defects indirectly influence craniofacial or nasal morphogenesis in *Odad3*-deficient embryos remains to be established [32,33]. One possible interpretation is that the E12.5 nasal phenotype represents an indirect consequence of altered early embryonic patterning. However, alternative explanations, including generalized developmental delay or previously unrecognized local effects of *Odad3* loss, cannot currently be excluded. In addition, because the E12.5 analysis was primarily qualitative, further morphometric assessment and rigorous developmental staging will be required to determine whether this represents a localized nasal defect or to rule out a generalized developmental delay.

The broader spatial distribution of the constitutive *Odad3^−/−^* phenotype compared to the adult-induced *Odad3^icKO^* model suggests that constitutive and adult-induced loss of *Odad3* have spatially and anatomically distinct consequences: while the conditional model primarily exhibits localized caudal defects, the constitutive absence of the gene disrupts the fundamental skeletal and mucosal scaffolding across the entire upper respiratory tract. The absence of detectable structural abnormalities in heterozygous (*Odad3^+/−^*) mice is consistent with haplosufficiency for the gross upper-airway phenotype under the conditions examined. However, molecular and functional analyses will be required to determine whether more subtle gene-dosage effects on ciliary motility or mucociliary clearance are present. Together with the previously reported reproductive phenotype during spermiogenesis [11], these observations raise the possibility of tissue-specific sensitivity to *Odad3* dosage.

The absence of detectable luminal material at E12.5 indicates that mucus accumulation emerges postnatally, after the developmental onset of the structural phenotype. Crucially, because the embryo develops in an environment sheltered from external airflow and pathogens, luminal secretions do not accumulate prior to birth. Following birth, impaired mucociliary clearance may promote secretion retention, which could in turn contribute to epithelial injury, inflammation, and progressive tissue remodeling. In constitutive mutants, these findings are consistent with a two-stage pathological process comprising an early developmental abnormality followed by postnatal mucus accumulation and tissue degeneration. In contrast, the adult-induced model demonstrates that post-developmental loss of *Odad3* is independently sufficient to produce an acquired caudal upper-airway phenotype.

Several limitations should be considered when interpreting these findings. First, the developmental stages were examined cross-sectionally using distinct genetic strategies; therefore, the constitutive and adult-induced models do not represent a single longitudinal disease trajectory. Second, the embryonic and early postnatal analyses were primarily qualitative, and contributions from generalized developmental delay or hydrocephalus-associated alterations in cranial growth cannot be excluded. Third, while systemic *Cre*-mediated recombination and target deletion in this inducible model were previously established [10,11], local recombination efficiency, protein loss, and functional ciliary motility (such as ciliary beat frequency) were not directly quantified in the nasal tissues of these archived imaging cohorts due to the technical requirements of PFA fixation and long-term EDTA decalcification optimized for 3D structural µCT mapping; therefore, the functional link between local *Odad3* loss, clearance failure, and CRS-like pathology remains inferential from previously published studies of airway defects in PCD mouse models [10,11]. Finally, although µCT and H&E analysis identified luminal material and extensive tissue remodeling, mucin-specific staining and inflammatory profiling will be required to define the composition of the accumulated material and confirm chronic inflammatory disease.

Looking forward, several avenues for future investigation emerge from this work. First, it will be imperative to decipher the specific molecular interactome of *Odad3* during the critical E12.5–E14.5 developmental window, exploring its potential involvement in key signaling pathways such as Hedgehog or Notch, which are known to govern epithelial patterning and turbinate outgrowth [34,35]. Second, to build directly upon the 3D structural baseline established here, future studies will incorporate tissue-specific molecular profiling (qPCR/IHC) and live ex vivo functional assays, such as high-speed video microscopy (HSVM) on fresh mucosal explants, to directly quantify local recombination, ciliary beat frequency (CBF), and beating kinematics following *Odad3* deletion. Additionally, ultrastructural transmission electron microscopy (TEM) on dedicated glutaraldehyde-fixed samples will serve to map axonemal dynein arm disruption at nanometer resolution. Third, the absence of an overt heterozygous structural phenotype motivates future dose–response and rescue studies to determine the minimum level of *Odad3* expression required to preserve upper-airway function [36]. Finally, combined with an independent gene-replacement or inducible rescue strategy, the *Odad3^icKO^* model could be used to test whether restoration of *ODAD3* function prevents or reverses established upper-airway pathology.

More broadly, recent comprehensive cryo-EM catalogs of the mammalian ciliary proteome have expanded the list of structural candidates well beyond the ~60 genes currently linked to PCD [37]. By cross-referencing these candidate genes with knockout lines available through the International Mouse Phenotyping Consortium (IMPC; https://www.mousephenotype.org/) [38], our decalcification-based µCT pipeline provides a high-content secondary screening framework to prioritize uncharacterized genes for functional validation. However, as illustrated by our findings in the *Odad3* model, constitutive ablation of essential axonemal proteins frequently leads to embryonic or perinatal lethality, often driven by severe hydrocephalus or laterality defects. Future screening applications should therefore favor conditional knockouts or adult-viable hypomorphic alleles to avoid systematically excluding essential structural genes. With these considerations, targeted µCT screening of IMPC ciliary mutants offers a tractable, orthogonal approach to connect structural proteomics with clinical genotype–phenotype correlations.

Collectively, our findings reveal distinct temporal consequences of *Odad3* loss in the murine upper airway. Constitutive disruption was associated with early abnormalities of nasal cavity morphogenesis that were detectable at E12.5 and accompanied by widespread structural alterations and luminal material accumulation after birth. By contrast, adult-induced deletion produced a predominantly caudal mucus-obstructive and degenerative phenotype, demonstrating that post-developmental loss of *Odad3* is sufficient to disrupt upper-airway homeostasis and cause PCD-like pathology.

These observations support the existence of both developmental and acquired components of *Odad3*-associated upper-airway disease. The combination of inducible genetic modeling, µCT-based virtual histology, and anatomically matched conventional histology provides a useful experimental framework for addressing these questions and for evaluating future therapeutic interventions.

## Data Availability

The original contributions presented in the study are included in the article/Supplementary Materials; further inquiries can be directed to the corresponding authors. Supplementary Materials and dataset are deposited on Zenodo at https://doi.org/10.5281/zenodo.21475754.

## Supplementary Materials

The following supporting information can be downloaded at https://zenodo.org/records/21475754 (accessed on 23 August 2026). Video S1: 3D virtual sectioning of nasal structure in adult WT mice; Video S2: 3D virtual sectioning of nasal structure in adult KO mice; Video S3: 3D virtual sectioning of nasal structure in constitutive heterozygous adult mice; Video S4: 3D virtual sectioning of nasal structure in tamoxifen induced heterozygous adult mice; Video S5: 3D virtual sectioning of nasal structure in early postnatal stage (11 days) WT mice; Video S6: 3D virtual sectioning of nasal structure in early postnatal stage (11 days) KO mice; Video S7: 3D virtual sectioning of nasal structure in WT mice embryos (E12.5); Video S8: 3D virtual sectioning of nasal structure in KO mice embryos (E12.5).

## Abbreviations

The following abbreviations are used in this manuscript:

PCD	Primary Ciliary Dyskinesia
Micro-CT	Micro-Computed Tomography
µCT	Micro-Computed Tomography
MCC	Mucociliary Clearance
ODA-DC	Outer Dynein Arm Docking Complex
IDAs	Inner Dynein Arms
ODAs	Outer Dynein Arms
CF	Cystic Fibrosis
2D	Two-Dimensional
3D	Three-Dimensional
WT	Wild-Type
NB	Nasal Bone
NS	Nasal Septum
ET	Ethmoturbinate
MT	Maxilloturbinate
NT	Nasoturbinate
VA	Atrioturbinate
MS	Maxillary Sinus
ES	Ethmoid Sinus
NLD	Nasolacrimal Duct
ND	Nasopharyngeal Duct
VNO	Vomeronasal Organ
H&E	Hematoxylin and Eosin staining
P11	Postnatal Stage 11 days
CRS	Chronic Rhinosinusitis
SPF	Specific Pathogen-Free
i.p.	Intraperitoneal
PFA	Paraformaldehyde
O.N.	Overnight
PBS	Phosphate-Buffered Saline
E12.5	Embryonic stage 12.5 days
OB	Olfactory Bulb
DMM	Dorsal Medial Meatus
SI	Superior Incisor
OE	Olfactory Epithelium
LM	Lamina Propria
TB	Turbinate Bone
NC	Primitive Nasal Cavity
NS	Cartilage primordium of the Nasal Septum
LV	Lateral Ventricle
FV	Follicles of Vibrissae
NP	Nasal Pit
OP	Olfactory nasal Placode
H	Heart
SC	Spinal Cord
